# Synthesis, Luminescent and Antibacterial Properties of Sol-Gel TiO_2_/TeO_2_/Nb_2_O_5_ Powders

**DOI:** 10.3390/ma18050946

**Published:** 2025-02-21

**Authors:** Kalina Ivanova, Albena Bachvarova-Nedelcheva, Reni Iordanova, Angelina Stoyanova, Petia Petrova, Lilia Yordanova, Iliana Ivanova

**Affiliations:** 1 Institute of General and Inorganic Chemistry, Bulgarian Academy of Sciences, Acad. G. Bonchev Str., Bl. 11, 1113 Sofia, Bulgaria; kalina@svr.igic.bas.bg (K.I.); reni@svr.igic.bas.bg (R.I.); 2 National Centre of Excellence Mechatronics and Clean Technologies, 8 Bul., Kl. Ohridski, 1756 Sofia, Bulgaria; 3 Department Chemistry and Biochemistry, Faculty of Pharmacy, Medical University-Pleven, Kl. Ohridski Str., 1, 5800 Pleven, Bulgaria; angelina.stoyanova@mu-pleven.bg; 4 Institute of Optical Materials and Technologies, Bulgarian Academy of Sciences, Acad. G. Bonchev Str., l.109, 1113 Sofia, Bulgaria; petia@iomt.bas.bg; 5 Faculty of Biology, Sofia University “St. Kliment Ohridski”, 8 Dragan Tsankov Blvd., 1164 Sofia, Bulgaria; lilijapj@uni-sofia.bg (L.Y.); iaivanova@biofac.uni-sofia.bg (I.I.)

**Keywords:** sol-gel, nanopowders, luminescence, photocatalytic properties, antibacterial properties

## Abstract

The present paper deals with the synthesis, characterization, and properties of sol-gel-derived TiO_2_/TeO_2_/Nb_2_O_5_ nanopowders. The gels were prepared using a combination of organic [Ti (IV) n-butoxide, Nb (V) ethoxide (C_10_H_25_NbO_5_)] and inorganic [telluric acid (H_6_TeO_6_)] precursors. The aging of gels was performed in air for several days in order to enable further hydrolysis. The phase formation of the gels was investigated by XRD upon heating in the temperature range of 200–700 °C. It was established that the gels heat-treated up to 300 °C exhibited a predominantly amorphous phase in all binary and ternary compositions. The amount of amorphous phase gradually decreased with increasing temperature, and the first TiO_2_ (anatase) crystals were detected at about 400–500 °C. The average crystallite size of TiO_2_ (anatase) in the powdered samples heat-treated at 400 °C was about 10 nm. By DTA, it was established that the decomposition of organics is accompanied by strong weight loss occurring in the temperature range of 200–300 °C. The completeness of the hydrolysis-condensation reactions was verified by IR and UV–Vis analyses. The UV–Vis spectra of the as-prepared gels exhibited red shifting of the cut-off. Photoluminescence spectra exhibited a change in intensity with varying temperature and composition. The performed photocatalytic tests showed that all powders possess photocatalytic activity toward Malachite green organic dye. The obtained nanopowders exhibited good antibacterial properties against *E. coli ATCC 25922*. The obtained samples can be considered as prospective materials for use as environmental catalysts.

## 1. Introduction

The sol-gel synthesis of Nb_2_O_5_-containing TiO_2_ powders has attracted scientific interest in the last decade due to their remarkable physicochemical properties, such as electrochromic behavior, photoelectric and photocatalytic activity, excellent chemical stability, and corrosion resistance in both acidic and alkaline media [1,2]. Various thin TiO_2_/Nb_2_O_5_ films have been developed that possess various innovative applications in photonics for improving the optical performance of different devices (optical filters), waveguide-based optical circuits, and transparent conductive electrodes [3,4,5]. Numerous papers are mainly devoted to the preparation of high-quality Nb_2_O_5_ thin films using relatively simple and inexpensive techniques, such as sol-gel and spin or dip coating [3,5]. Obviously, the sol-gel method still attracts scientific attention because of its versatility, low cost, and low-temperature processing [6,7]. Additionally, it allows control of the microstructure of the coating and produces durable and chemically stable films. Moreover, the versatility of the sol-gel process in preparing porous film has an additional advantage to be exploited.

Recently, many studies have been conducted on the use of Nb_2_O_5_ nanoparticles for environmental remediation in water through photocatalytic processes. Regarding this, Nb_2_O_5_ showed great potential because of its stability in an aqueous medium, its surface acidity, and its redox and photocatalytic properties. Previous studies on Nb_2_O_5_-doped TiO_2_ powders also reported on dye degradation as well as the degradation of aromatic compounds [8,9]. Sedneva et al. [10] studied the effects of Nb amount and calcination temperature on photoactivity, phase composition, and crystallite size. Despite these papers on the Ti-Nb combination reported in the literature [11,12], investigations on the TiO_2_/N_2_O_5_ system have not been exhausted yet. There is still a lack of studies about the impact of the synthesis method on the properties of the obtained materials. It is also not clear what the effect of pH is on the photocatalytic activity of the oxides, as it is known that this factor directly affects the surface charges of the catalyst and the potential of the valence and conduction bands [13,14].

There are scarce data concerning the antibacterial activity of Nb_2_O_5_-containing sol-gel-derived powders. It was found that TiO_2_ + Nb_2_O_5_ films exhibited antibacterial potential against *S. mutans* [15]. It is also known that Nb_2_O_5_ is very important for the bioactivity of glasses. The antibacterial studies of CaF_2_–CaO–B_2_O_3_–P_2_O_5_–SrO glasses doped with different Nb_2_O_5_ concentrations found that they are effective toward Gram-negative *Escherichia coli* (*E. coli*) and Gram-positive *Staphylococcus aureus* (*S. aureus*) bacteria [16].

Our group has experience in the sol-gel synthesis of composite powders containing TiO_2_, and we have so far produced many binary, ternary, and multicomponent composites with strong photocatalytic and antibacterial qualities [17,18,19,20,21]. This work is an extension of our earlier studies on the sol-gel synthesis of powdered TiO_2_ nanocomposite. Since the emphasis is now on compositions with enhanced photocatalytic and antibacterial capabilities, the search for novel combinations of compositions that have not yet been studied has been expanded. To the best of our knowledge, there are no reported data on TiO_2_/TeO_2_/Nb_2_O_5_ sol-gel-derived powders and no studies of their antibacterial and photocatalytic applications, which underlines the novelty in the current work.

In the present paper, we study the synthesis and luminescent and antibacterial properties of TiO_2_/TeO_2_/Nb_2_O_5_ powders obtained by the sol-gel method. Their photocatalytic activity has also been verified.

## 2. Materials and Methods

### 2.1. Gelling and Drying

**Figure 1:**
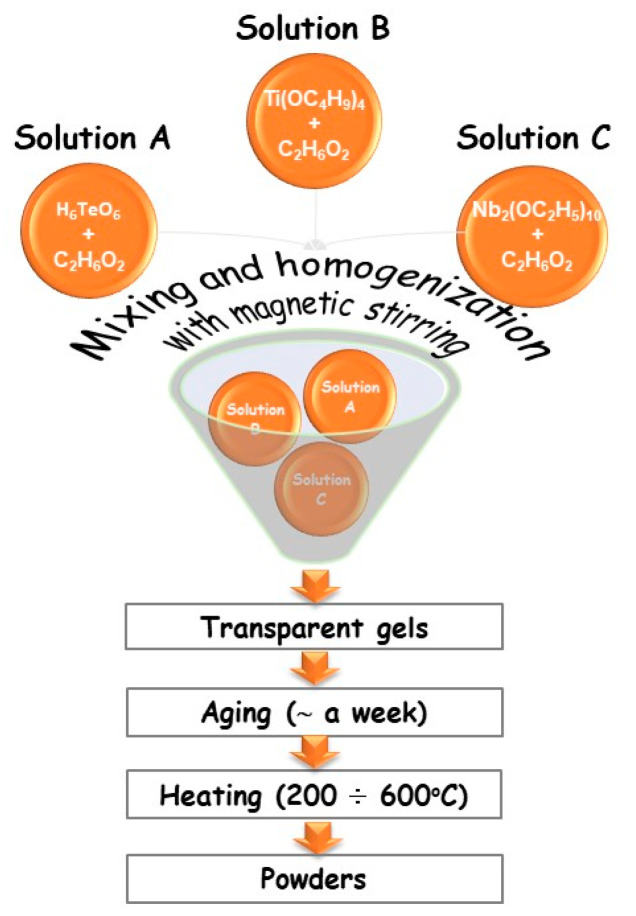
Scheme for the sol-gel synthesis of the samples.

Based on our previous findings on gel formation in other ternary TiO_2_–TeO_2_–MnOm (B_2_O_3_, ZnO, SeO_2_) systems [19,20,21], compositions containing higher TiO_2_ content were selected—80TiO_2_/20Nb_2_O_5_, 80TiO_2_/10TeO_2_/10Nb_2_O_5_, and 50TiO_2_/25TeO_2_/25Nb_2_O_5_—denoted as samples A, B, and C, respectively. They were subjected to detailed investigations. The gels were prepared using a combination of Te(VI) acid (99.99%, Aldrich, St. Louis, MO, USA), along with Ti butoxide(IV) (≥99%, Fluka AG, Buchs, Switzerland) and niobium(V) ethoxide (C_10_H_25_NbO_5_) (Merck, Darmstadt, Germany) as precursors dissolved in ethylene glycol (C_2_H_6_O_2_) (99%, Aldrich) (Figure 1). Telluric acid (H_6_TeO_6_) was chosen to overcome the problem with the high hydrolysis rate of tellurium(VI) alkoxide, which has been analyzed in numerous papers [20,21]. The precursor solutions were subjected to 5–10 min of intensive stirring at room temperature to achieve complete dissolution. No direct addition of water was made to the precursor solutions. The sol-gel hydrolysis reaction was acquired from absorbed atmospheric moisture. The measured pH was 4–5, depending on composition. The gelation for the investigated compositions occurred immediately. To complete the hydrolysis, aging of the gels was performed in air for several days. The obtained gels were subjected to stepwise heating from 200 to 600 °C for one hour of exposure time in air. Aiming to verify the phase and structural transformations of the gels, heating in the range of 200–600 °C until powders were obtained was performed. The selection of the temperatures was made based on our previous investigations [20].

### 2.2. Sample Characterization

Powder XRD patterns were registered at room temperature with a Bruker D8 Advance (Berlin, Germany) X-ray powder diffractometer with a Cu Ka radiation (k = 1.54056 Å) with a LynxEye solid position sensitive detector and X-ray tube operated at 40 kV and 40 mA. X-ray diffraction patterns were recorded in the range of 5.3–80° for 2 h with a step of 0.02° 2 h. Differential thermal analysis (LABSYSTM EVO apparatus, Setaram, Lyon, France) with a Pt-Pt/Rh thermocouple at a heating rate of 10 K/min in air flow and with Al_2_O_3_ as a reference material was used to study the decomposition process of the gels. The accuracy of the temperature was ±5 °C, and the heating of the samples was limited up to 700 °C. Gases evolved (EGA) during the thermal treatments were analyzed by mass spectrometry (MS) with a Pfeiffer OmniStar^TM^ mass spectrometer (Pfeiffer Vacuum Technology AG, Wetzlar, Germany). Mass spectra recorded for the investigated samples show the m/z = 14, 18, and 44 signals, being ascribed to CH_2_, H_2_O, and CO_2_, respectively. The infrared spectra were registered in the range of 1600–400 cm^−1^ using the KBr pellet technique on a Nicolet-320 FTIR spectrometer (Madison, WI, USA) with 64 scans and a resolution of ±1 cm^−1^. A UV–Vis diffused reflectance spectrophotometer Evolution 300 (Thermo Electron Corporation, Madison, WI, USA) with a magnesium oxide reflectance standard as the baseline was used for recording the optical absorption spectra of the powdered samples in the wavelength range of 200–800 nm. Planck’s equation was applied for the determination of the absorption edge and optical bandgap (Eg) [22,23,24]. Photoluminescence spectra of the powders were measured at an excitation wavelength of 325 nm in the range of 340–640 nm using a Fluorolog-3 spectrofluorometer (Horiba/Jobin-Yvon, Longjumeau, France). The specific surface areas (BETs) were determined by low-temperature (77.4 K) nitrogen adsorption in a NOVA 1200e surface area and pore analyzer (Quantachrome, Boynton Beach, FL, USA) at relative pressures p/p0 = 0.1–0.3 using the BET equation.

#### 2.2.1. Photocatalysis

The photocatalytic performance of the synthesized TiO_2_-containing samples was evaluated by using Malachite green (MG) as a representative dye pollutant. All photodegradation experiments were carried out with a 5 ppm aqueous solution of MG at room temperature. In each run, 100 mg of TiO_2_-based sample was suspended in 150 mL of a solution of MG and sonicated for 10 min. Prior to irradiation, the suspensions were magnetically swirled in the dark for 30 min to ensure the establishment of an adsorption–desorption equilibrium at the sample surface. Then, the reaction mixtures were illuminated with a UV lamp (Sylvania BLB 50 Hz 8 W T5, emitting primarily at 365 nm) fixed at a distance of 10 cm above the solution’s surface. During illumination, the dispersions were continuously stirred in order to ensure satisfactory mixing of the reacting particles in the mixtures. Before and at regular intervals during the irradiation, aliquots of 3 mL of the suspensions were withdrawn and centrifuged (5000 rpm) for 10 min. The residual concentration of MG dye in the supernatants was determined by measuring the absorbance using a UV–Vis spectrophotometer (Genesys 10 S) at λ_max_ = 618 nm. The percentage of residual MG dye is expressed as C/C_0_ × 100%, where C_0_ is the concentration of MG at zero time, and *C* is its concentration at time *t* of illumination.

#### 2.2.2. Test of Antibacterial Activity

All investigated samples heat-treated at 400 and 600 °C were verified for antibacterial activity against *E. coli*. The antimicrobial effect was tested on the Gram-negative bacteria *Escherichia coli* ATCC 25922, supplied by the National Bank of Industrial microorganisms and cell cultures (NBIMCC), Bulgaria. A spot test assay was performed at the 3rd and 24th hours to assess the antimicrobial activity of the nanopowder dispersions. The broth microdilution method was used to determine the minimal bactericidal (MBC) and minimal inhibitory concentration (MIC).

The bacterial suspension was adjusted to a turbidity of 0.5 McFarland (10^8^ colony-forming units per milliliter) using a densitometer. To determine the MIC and MBC, a 96-well plate was used. Each well was dripped with 100 µL of bacterial suspension and 100 µL of nanopowders dispersed in deionized water at different concentrations (35, 25, 17.5, 15, 12.5, 10, 6, 3, and 1.5 mg/mL) after sonication for 15 min in an ultrasonic bath by Sonorex, Bandelin (Germany). For every tested sample, a separate control was prepared that only consisted of bacterial suspension. Then, the 96-well plate was incubated overnight (20–24 h) at a temperature of 35 ± 2 °C.

At the 3rd and 24th hour from the incubation of the well plate, a spot test was prepared: 5 μL samples from each well were plated onto solid media and incubated at the same temperature. Bacterial growth in each spot was evaluated on the following day, indicating the inhibitory or bactericidal effect of the samples used. To quantify viable bacteria, tenfold serial dilutions were prepared based on the growth results from the 3rd-hour spot test and inoculated onto Mueller–Hinton agar plates.

The MIC was defined as the lowest concentration of the sample that inhibited the growth of *Escherichia coli*, while the MBC was determined as the lowest concentration of the sample that achieved a ≥99.9% reduction in colony-forming units per milliliter (CFU/mL). The concentration of surviving treated bacteria is calculated using the following formula: CFU/mL = (number of colonies × dilution factor)/volume of the inoculum sample.

## 3. Results and Discussion

### 3.1. XRD and DTA Analyses

**Figure 2:**
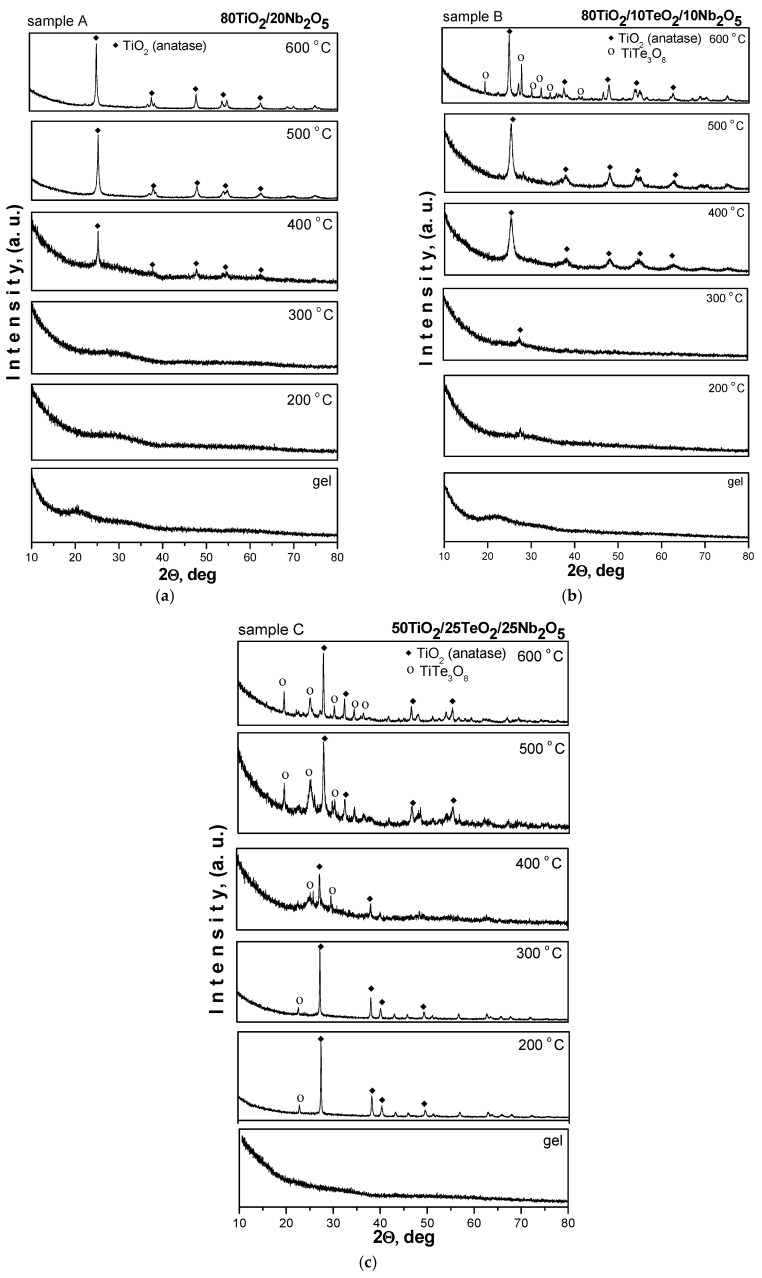
XRD patterns of investigated samples (**a**) 80TiO_2_/20Nb_2_O_5._ (**b**) 80TiO_2_/10TeO_2_/10Nb_2_O_5_. (**c**) 50TiO_2_/25TeO_2_/25Nb_2_O_5_.

The crystallinity and phase formation of the investigated samples calcined at different temperatures (200–600 °C) were analyzed by XRD. The XRD patterns of the samples are shown in Figure 2. As seen in the figure, the samples containing 80 mol% TiO_2_ preserved the amorphous state up to 300 °C, while in the third sample (sample C, 50 mol% TiO_2_), first TiO_2_ (anatase, JCPDS 78-2486) along with TiTe_3_O_8_ (JCPDS 50-0250) crystals are already detected at 200 °C and are present up to 300 °C. The main peculiarity in the XRD pattern of sample C is that the amount of crystalline TiTe_3_O_8_ phase increased with increasing temperature. In the other two samples, A and B, the first anatase crystals appeared at 400 °C, and this remained the dominant phase up to 600 °C in both samples. Similar to the ternary sample C, the XRD pattern of sample B exhibited the presence of TiTe_3_O_8_ at 600 °C, but that phase appeared only at the highest temperature, while in sample C, the same phase coexisted with the TiO_2_ (anatase) in the temperature range of 400–600 °C. The average crystallite size of the TiO_2_ (anatase) particles, calculated from the broadening of the diffraction line using Sherrer’s equation, is in the range of 10–30 nm, as the XRD pattern of sample B at 400 °C exhibited a broader peak than the other samples. The obtained XRD data are very similar to those reported in other papers [25,26].

### 3.2. DTA Results

**Figure 3:**
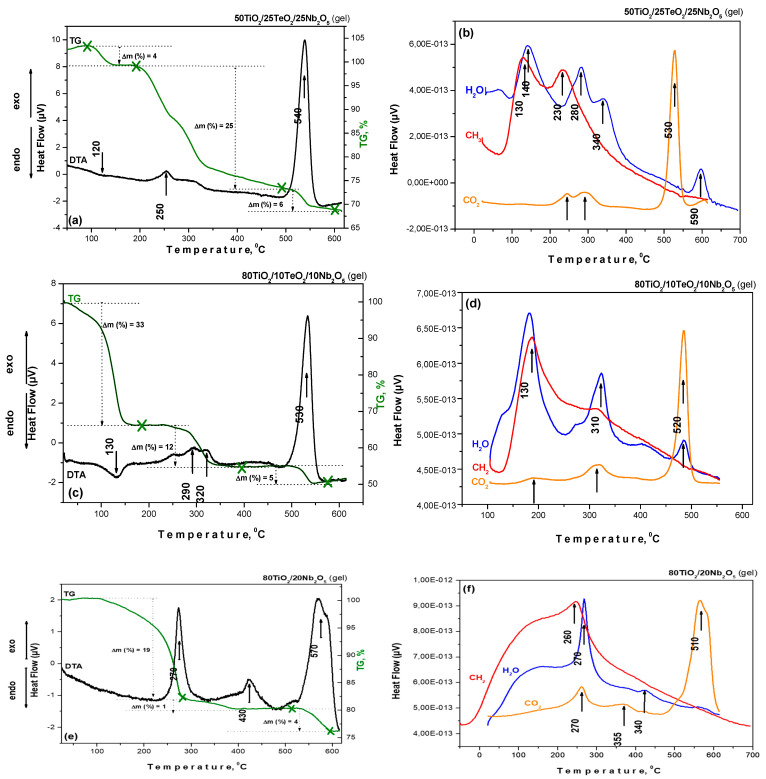
DTA/TG curves (**a**,**c**,**e**) and mass spectra (**b**,**d**,**f**) of the investigated compositions.

The thermal decomposition data of investigated gels in air are shown in Figure 3. As seen, all gels are characterized by endothermic peaks in the range of 100–200 °C, with weight loss between 8 and 18% depending on composition. The DTA curves of binary gel A differ from those of the ternary ones (B and C). Generally, the evaporation of organic solvent and the local removal of water that has formed a connection with the material’s surface are the primary causes of mass loss. Upon examining the DTA/TG curves, several exothermic peaks may be seen throughout the 220–440 °C range. The first one is observed in the 250–290 °C range for all investigated gels, which is related to the decomposition of Ti (IV) butoxide and niobium (V) ethoxide. It has to be noted that this peak is stronger for binary sample A, and its low intensity in the ternary samples could be attributed to the presence of TeO_2_, which delays the decomposition of the metal alkoxides. The observed weight loss exhibited the highest value of about 20% in sample A, which, according to the TG curve, finished at nearly 420 °C. The second exothermic peak differs for the binary and ternary samples—for sample A, it is about 430 °C, while for the ternary ones, it is near 320 °C (Figure 3). This peak could be attributed to the crystallization of TiO_2_ (anatase), and it is accompanied by a strong weight loss for the ternary samples (12 and 25%). The last exothermic peak is at about 570 °C for sample A and ~530–540 °C for the ternary samples (B and C), and the calculated weight loss is very low, ~5% in all samples. The calculated total weight loss for the investigated samples is below 50%. For comparison, other authors have stated similar temperatures for DTA investigations [10].

### 3.3. Spectroscopic Characterization

#### IR Investigations

**Figure 4:**
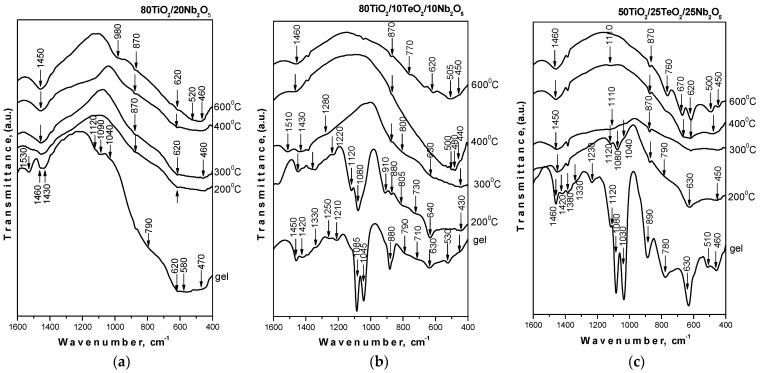
IR spectra of investigated samples heat-treated at different temperatures: (**a**) 80TiO_2_/20Nb_2_O_5_, (**b**) 80TiO_2_/10TeO_2_/10Nb_2_O_5_, and (**c**) 50TiO_2_/25TeO_2_/25Nb_2_O_5_.

The IR spectra of the samples heated at different temperatures are shown in Figure 4. The vibration band assignments were made based on spectral data discussed in our previous investigations on sol-gel-derived binary and ternary composite powders containing TiO_2_ [27,28]. As seen in the figure, the IR spectra of the binary sample 80TiO_2_/20Nb_2_O_5_ differ from those of the ternary ones. Generally, two main regions could be distinguished in the IR spectra of the gels: 1500–1030 cm^−1^ and 900–400 cm^−1^. The similarity with our previous works is expressed by the presence of bands at 1120, 1090–1080, and 1040–1030 cm^−1^ that could be assigned to the Ti-O-C stretching vibrations contributing to the formation of a mixed organic–inorganic amorphous structure. The position of these bands provides additional information for the determination of the degree of hydrolysis [29,30]. On the other hand, the bending vibrations of CH_3_ and CH_2_ groups are also situated in this region [31,32]. As seen in the figure, the low intensity of absorption bands in the 1120–1040 cm^−1^ region is an indication of more completed hydrolysis-condensation processes. Additionally, the obtained results show that the intensity of these bands decreased in the following order: sample C > sample B > sample A. The lowest intensity is observed in the spectrum of 80TiO_2_/20BNb_2_O_5_ (sample A) as compared to the ternary compositions, which indicates that niobate units are incorporated in the titanate network and lead to a greater degree of hydrolysis in the gel and the sample heated at 200 °C. Obviously, the decrease in the TiO_2_ content and addition of TeO_2_ retard the hydrolysis-condensation processes, as was observed in the spectrum of sample C in comparison to the other two samples.

The spectral behavior of the samples in the range of (300–600 °C) is similar, and only vibrations of inorganic structural units could be seen (Figure 4). These bands are low-intensity and broadened, which is typical for disordered systems. In all compositions, the observed bands in the region of 700–400 cm^−1^ are related to the vibrations of a Ti-O-Ti network [33,34,35]. On the other hand, the ternary compositions exhibited bands at 670 and 620 cm^−1^, which are assigned to the vibrations of TeO_4_ trigonal bipyramids (tbps) [34,35]. At higher temperatures (600 °C), these bands become stronger when the Nb_2_O_5_ content is 25 mol%, as seen in the IR spectrum of sample C, which has the highest TeO_2_ content (25 mol%). This phenomenon was found by Kabalci et al. [35], who investigated the structure of TeO_2_/Nb_2_O_5_/TiO_2_ glasses. Obviously, there are strong overlaps in the 750–400 region, which hinders the more precise assignments of the inorganic bands. The infrared band at 870 cm^−1^ can be ascribed to the ν_1_ stretching vibration of short Nb–O bonds in isolated NbO_6_ octahedra [27,36]. The IR analysis demonstrated that the organic–inorganic amorphous phase obtained by the sol-gel method entirely changed into an inorganic one above 400 °C. The results obtained correlate well with the XRD data already discussed above, as well as with our preceding investigations on obtaining TiO_2_-containing compositions by the sol-gel method.

### 3.4. Diffuse Reflectance Spectroscopy (DRS) in the UV–Visible Region

**Figure 5:**
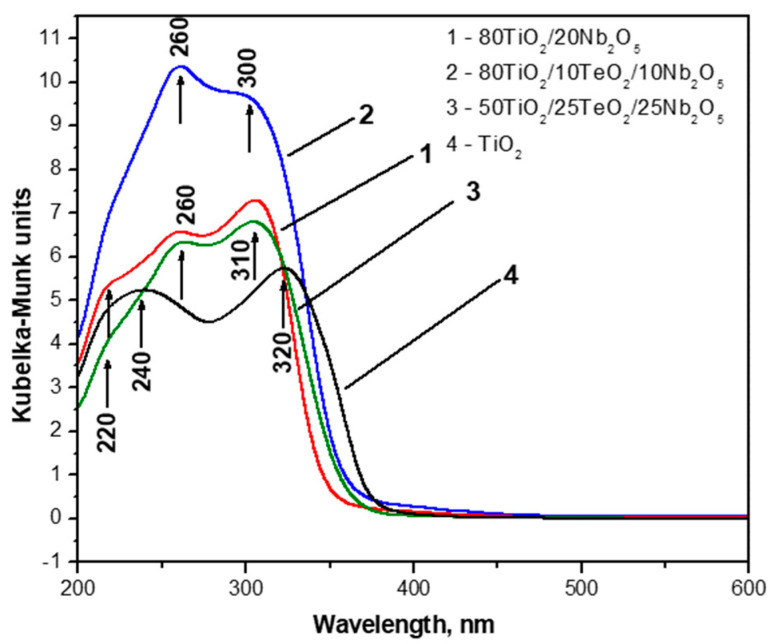
UV–Vis spectra of investigated samples compared with Ti(IV) butoxide gel and pure TiO_2_.

The optical absorption spectra of investigated gels were compared to those of TiO_2_ obtained by Ti(IV) butoxide gel (Figure 5). The absorption edge and optical band gap values of the samples are summarized in Table 1. All gels possess good absorption in the UV region, where the ternary sample 80TiO_2_/10TeO_2_/10Nb_2_O_5_ exhibited the highest absorption, while sample C—which had the lowest TiO_2_ content—showed a decrease in the absorption. This observation might be associated with interface charge transfer between TiO_2_ and Nb_2_O_5_ [37,38].

The UV–Vis spectra exhibited two maxima at 230–260 nm and 300–325 nm, which could be ascribed to the isolated TiO_4_ and TiO_6_ units, respectively [39]. It is seen that the TiO_2_ gel showed comparable intensity of UV peaks at 240 nm and 320 nm, which is an indication of the commensurate amount of TiO_4_ and TiO_6_ polyhedra in the gel network. The intensities of these bands in the other gels vary depending on composition. The UV–Vis spectra were also used to determine the optical band gap (E_opt_) of the investigated samples (Table 1). In all absorption spectra, the absorption edge values of the Nb_2_O_5_-containing gels were calculated between 348.89 and 363.2 nm. This shows a blue shift for these powders in comparison to the pure TiO_2_ gel. The estimated band gaps of the investigated gels are in the range of 3.41 to 3.55 eV, which is similar to that reported in the literature [37,40,41]. According to the literature, the band gap of pure Nb_2_O_5_ is 3.4 eV as a typical n-type wide-bandgap semiconductor, and the obtained values are very close to that [42]. Some authors have stated that the wavelength and intensity of Nb_2_O_5_ absorption spectra depend on the size, crystalline type, morphology, and method of synthesis. The smaller size of Nb_2_O_5_ particles leads to blue shifting in the absorption spectra [40,43].

### 3.5. Luminescent Properties

**Figure 6:**
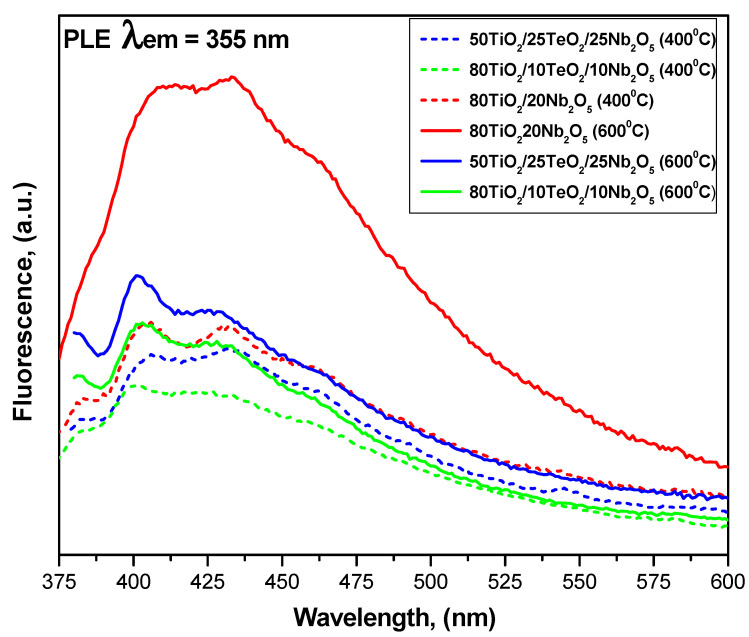
Room-temperature emission PL spectra of investigated samples heated at 400 and 600 °C.

Room-temperature emission PL spectra of the investigated samples heated at 400 and 600 °C are shown in Figure 6. Two obvious signals at 400 and 430 nm are observed, originating from the irradiative recombination of free electrons in shallow traps and sub-bands below the conductive band and free holes at the valance band edge [44,45,46]. The shape of the spectra is similar for all samples, indicating that the addition of different TeO_2_ and Nb_2_O_5_ amounts to TiO_2_ does not induce new emission phenomena. It is obvious that the samples heat-treated at a higher temperature (600 °C) exhibited stronger PL signals. As mentioned above, the intensity of the spectra results from both the recombination of electron–hole pairs (the lower the intensity, the lower the recombination) and the presence of lattice defects or oxygen vacancies (the higher the intensity, the greater their number) [47,48,49]. Generally, the intensities of the two PL signals are reduced in samples containing lower Nb_2_O_5_ amounts, except those in sample A, which did not contain TeO_2_. Perhaps its presence decreases the recombination rate of photogenerated electron–hole pairs. Therefore, the binary 80TiO_2_/20Nb_2_O_5_ is expected to exhibit promoted activity in photocatalytic tests. It is also noticed that samples containing 20 and 25 mol% Nb_2_O_5_ showed stronger signals at 400 nm, which is probably due to the presence of oxygen vacancies [50]. For all samples, the main emission band is around 400 nm, and according to Tam et al. [51], this can be attributed to the indirect band-to-band transition of electrons in TiO_2_. On the other hand, this short-wavelength PL could be ascribed to the near-band gap emission of Nb_2_O_5_ [1,50]. The other PL emission band (430 nm) could be ascribed to the structure defects [1,40].

### 3.6. Photocatalytic Properties

**Figure 7:**
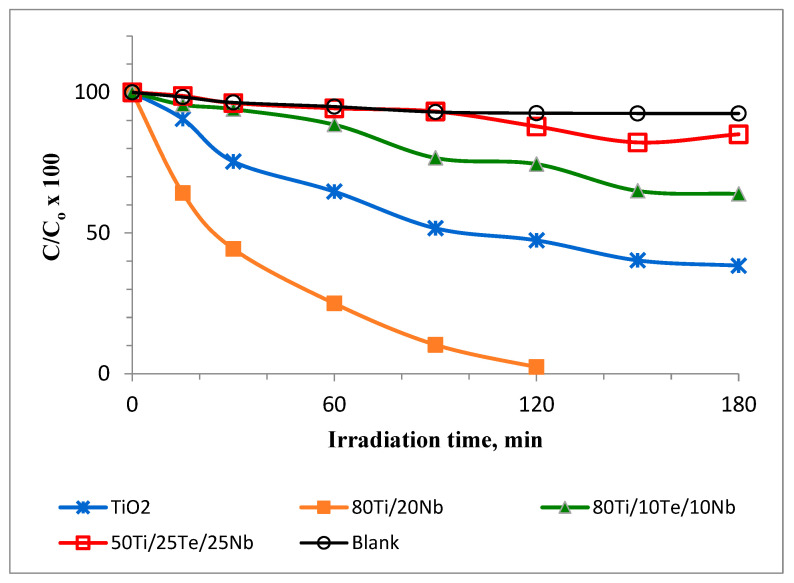
Photocatalytic properties of investigated samples.

The photocatalytic activity of the as-synthesized TiO_2_ samples was tested by the degradation of MG under UV irradiation. Figure 6 presents the results of photocatalytic experiments with the investigated samples compared with those of pure TiO_2_ synthesized from Ti(IV) butoxide. As can be seen, the photocatalytic performance of the sample 80TiO_2_/20Nb_2_O_5_ was more efficient than the other assayed samples toward MG degradation (Figure 7). After 120 min of UV irradiation, the MG dye was completely decolorized by sample A (80TiO_2_/20Nb_2_O_5_), while for samples B, C, and pure TiO_2_, the percentages of residual MG were about 75%, 88%, and 50%, respectively. The BET measurements provide additional confirmation of the photocatalytic results of the species. The specific surface area (SSA) of the ternary samples is about 35–45 m^2^/g, while the binary one exhibited a value of 70 m^2^/g.

A literature survey [51] summarized that the probable mechanism for the discoloration of MG dye could be explained as follows: excited surface electrons interact with the dissolved oxygen molecule to form superoxide radicals, and holes oxidize water to produce highly active hydroxyl radicals while allowing nanoparticles to interact with the ionic dye. Regarding the fundamental constituents, the chromophores responsible for characteristic colors in MG were broken down; thus, the dye degraded in the presence of powders under UV light illumination.

### 3.7. Antibacterial Properties

Aiming to obtain more complete information concerning the antimicrobial activity of the prepared samples, two kinds of tests—quantitative (microdilution method) and qualitative (spot test) were performed. The performed spot test was used to predict the antibacterial activity of the as-obtained samples. Bearing in mind the obtained data, only results from the quantitative analysis have been presented. Moreover, it should be noted that the antibacterial activity of all samples was studied without UV light, as in the Refs. [51,52].

**Figure 8:**
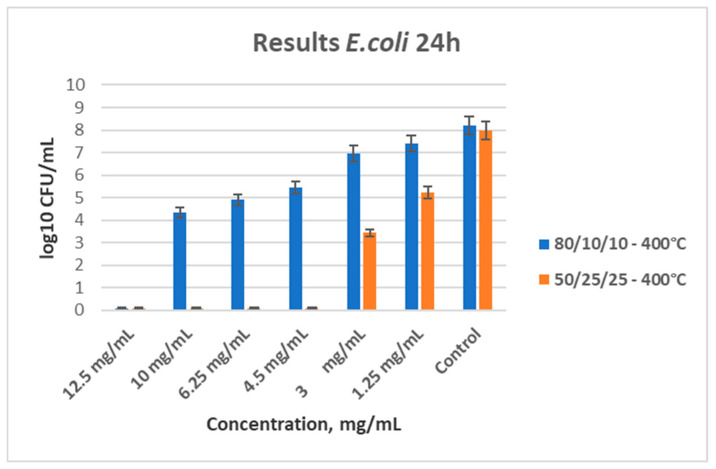
Antibacterial effect of both ternary samples heat-treated at 400 °C, with different concentrations influencing *E. coli*.

As shown in Figure 8, both ternary samples heated at 400 °C demonstrated antibacterial properties, and their MBC and MIC were determined. The MBC for 80TiO_2_/10TeO_2_/10Nb_2_O_5_ (sample B) is 12.5 mg/mL, and the MIC is 10 mg/mL. For sample C (50TiO_2_/25TeO_2_/25Nb_2_O_5_), the MBC stands at 4.5 mg/mL.

**Figure 9:**
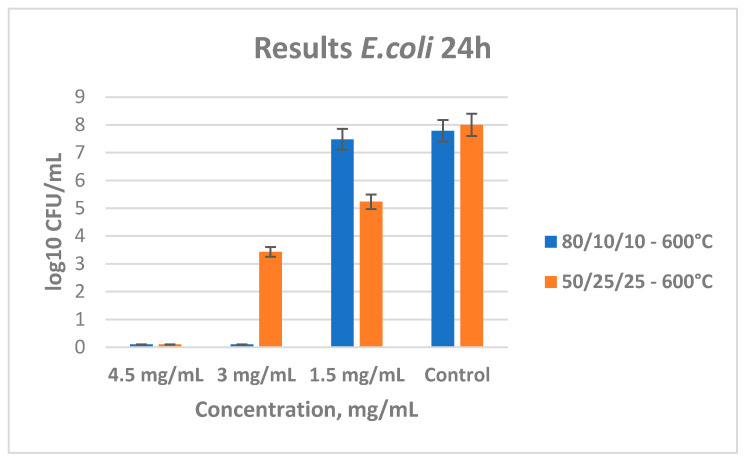
Antibacterial effect of ternary samples (B and C) heat-treated at 600 °C with different concentrations influencing *E. coli*.

Figure 9 shows the results from the tested antibacterial properties of the two ternary samples heated at 600 °C. Again, their MBC and MIC were determined with the broth microdilution method. Sample B shows a good antimicrobial effect, and the minimal bactericidal concentration is determined to be 3.0 mg/mL. For sample C, the MBC value was found to be 4.5 mg/mL. Obviously, there is an increase in the minimal bactericidal concentration for sample C, which is probably due to the decreased content of titania.

**Figure 10:**
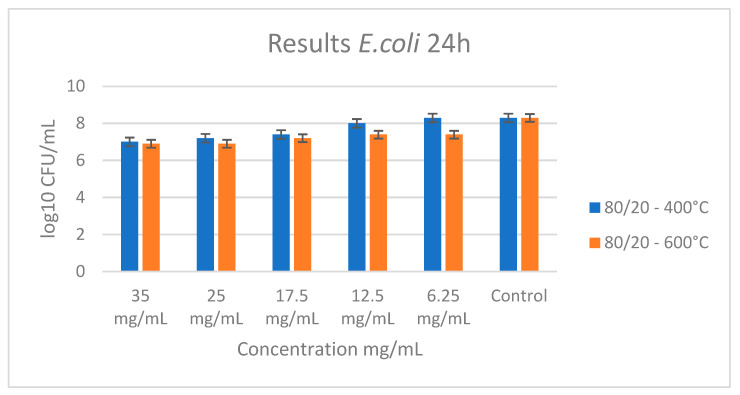
Antibacterial effect of sample A (80TiO_2_/20Nb_2_O_5_) heat-treated at 400 °C and 600 °C with different concentrations influencing *E. coli*.

The tested binary composition did not show a bactericidal effect with the following concentrations: 35, 25, 17.5, 12.5, and 6.25 mg/mL. As shown in Figure 10, a slightly bacteriostatic effect for the sample heated at 600 °C at a concentration of 35 mg/mL was observed. The absence of bactericidal activity in the binary sample further supports the significant role of tellurium in antimicrobial action. Obviously, the observed antibacterial activity of our powders can be attributed to the synergetic effect of anatase, TeO_2_, and Nb_2_O_5_. The plate count method was used to determine the quantity of treated bacteria. The bacterial count in the control sample was measured at 10^8^ CFU, while the treated sample showed a reduction in bacterial count to 10^6^ CFU. This demonstrated a 100-fold decrease in colony count in the treated sample compared to the control.

It is well known that antibacterial activity can be explained by the nature of titanium dioxide, and one of the main mechanisms of its action is through the generation of reactive oxygen species (ROS) on its surface. This process can occur even in the absence of photoactivation [51]. It has been reported that an electromagnetic attraction between microorganisms and the TiO_2_ NPs is due to the positive charge carried by the TiO_2_ and the negative charges carried by the microorganism’s surface, leading to oxidation reactions. Generally, in this way, TiO_2_ deactivates cellular enzymes and DNA by coordinating with electron-donating groups, such as thiols, carbohydrates, amides, indoles, and hydroxyls. As a result, the bacterial cell walls lead to increased permeability and cell death [52].

## 4. Conclusions

The sol-gel method was used for the preparation of binary and ternary TiO_2_/TeO_2_/Nb_2_O_5_ gels. By using XRD and IR analysis, it was determined that niobium oxide retained the organic components up to 300 °C and that above 400 °C, the organic–inorganic amorphous phase entirely changed into an inorganic one. Applying UV–Vis spectroscopy, two maxima relating to the isolated TiO_4_ units and condensed TiO_6_ groups were observed at about 230–260 nm and 305–325 nm, respectively. The photoluminescence spectra exhibited a change in intensity with varying temperatures and TeO_2_ amounts. The photocatalytic tests showed that the binary sample possesses superior photocatalytic activity toward Malachite green organic dye. The two ternary samples exhibited good antibacterial properties against *E. coli ATCC 25922*, but the best one was 80TiO_2_/10TeO_2_/10Nb_2_O_5_ heat-treated at 600 °C. The obtained results reveal that the latter could be used as an antibacterial agent.

## Figures and Tables

**Table 1:** Cut-off and optical band gap values (Eg) of the investigated gel compositions.

Composition (mol%)	Cut-Off (nm)	Eg (eV)
Ti(IV) n-butoxide	389.71	3.18
80TiO_2_/20Nb_2_O_5_	348.89	3.55
80TiO_2_/10TeO_2_/10Nb_2_O_5_	363.2	3.41
50TiO_2_/25TeO_2_/25Nb_2_O_5_	354.58	3.49

## Data Availability

The original contributions presented in this study are included in the article. Further inquiries can be directed to the corresponding author.
